# Polyvinyl chloride-related insulin adsorption in critical care: a narrative review with implications for patient safety and green hospital reform

**DOI:** 10.3389/fmed.2026.1718431

**Published:** 2026-04-29

**Authors:** Selwan Hamza Elgazzar, Rasha Kadri Ibrahim, Yasmin Moustafa Ayoub, Raeed Alanazi, Abdulaziz K. Alanazi, Hyam Refaat Tantawi, Ahmad R. Al-Qudimat, Ashwag Alsharidah, Abdelaziz Hendy

**Affiliations:** 1 Nursing Education Administration, King Saud Medical City, Riyadh, Saudi Arabia; 2 Nursing Department, Fatima College of Health Sciences, Al Dhafra Region, Madinat Zayed, United Arab Emirates; 3 King Saud Medical City, Riyadh, Saudi Arabia; 4 Department of Nursing Administration and Education, College of Nursing, King Saud University, Riyadh, Saudi Arabia; 5 Nursing Administration, King Saud Medical City, Riyadh, Saudi Arabia; 6 Department of Pediatric Nursing, Faculty of Nursing, Ain Shams University, Cairo, Egypt; 7 Surgical Research Section, Surgery Department, Hamad Medical Corporation, Doha, Qatar; 8 QU Health, Qatar University, Doha, Qatar; 9 Department of Physiology, College of Medicine, Qassim University, Buraydah, Saudi Arabia; 10 Department of Maternal and Child Health Nursing, College of Nursing, Qassim University, Buraydah, Saudi Arabia; 11 Department of Pediatric Nursing, Faculty of Nursing, Ain Shams University, Cairo, Egypt

**Keywords:** critical care, DEHP, diabetes, green hospital, insulin adsorption, intravenous insulin, nursing, patient safety

## Abstract

**Introduction:**

Insulin infusion therapy is critical for glycemic management in intensive care units (ICUs), yet its effectiveness is compromised by adsorption to infusion materials, particularly polyvinyl chloride (PVC) tubing and bags plasticized with di(2-ethylhexyl) phthalate (DEHP). This phenomenon results in underdosing, unstable glucose control, and increased risk of hypo- and hyperglycemia. At the same time, PVC and DEHP raise environmental and toxicological concerns, driving global efforts toward green hospital reforms.

**Aim:**

This narrative review synthesizes current evidence on insulin adsorption across PVC and DEHP-free infusion systems, evaluating implications for clinical safety, glycemic control, and sustainable healthcare practices.

**Methods:**

A Structured Narrative Analysis Review (SNAR) was conducted, with systematic searches in eight databases guided by PICO and SPIDER frameworks. Thirty-eight studies met the inclusion criteria. Data were thematically analyzed using Braun and Clarke’s six-phase method.

**Results:**

Insulin adsorption varied by material, with PVC demonstrating the highest losses (up to 70%), particularly at low flow rates and during the early infusion period. Alternatives such as polyethylene (PE), polypropylene (PP), and fluorinated ethylene propylene (FEP) showed significantly lower adsorption, while SEBS and TPO offered partial improvements but did not eliminate losses. Priming or preconditioning infusion sets with 20 mL of insulin solution consistently minimized early adsorption. Thematic analysis revealed four main domains: (1) insulin adsorption as a clinical safety risk, (2) material-dependent variability, (3) mitigation strategies, and (4) sustainability implications.

**Conclusion:**

Insulin adsorption on PVC infusion systems is a clinically significant barrier to precise glycemic control in critical care. Evidence supports the use of priming techniques and alternative materials (PE, PP, FEP) to reduce losses. Transitioning toward DEHP-free and non-PVC devices aligns with green hospital reforms, offering dual benefits for patient safety and environmental sustainability.

## 1 Introduction

Intravenous insulin therapy is essential for glycemic management in critically ill patients, as optimal blood glucose levels are directly linked to improved clinical outcomes. In some situations, the effectiveness of insulin delivery can be affected by the drug sticking to the materials used for infusion. Polyvinyl chloride (PVC) is commonly used in intravenous (IV) tubing and bags. It is well-known for its ability to adsorb insulin onto its surfaces, especially when it is plasticized with di(2-ethylhexyl) phthalate (DEHP). This adsorption could lead to a significant loss of insulin during injection, which could mean not getting enough of it, not controlling blood sugar levels well enough, and serious safety issues for patients.

The application of medical devices containing PVC presents several issues, particularly with their insulin absorption efficacy, which significantly impacts the effectiveness of insulin therapy. Patients may encounter challenges in obtaining their insulin if it adheres to PVC surfaces. This may cause fluctuations in blood sugar levels, increasing the likelihood of hypoglycemia. Consequently, insulin dosages must be adjusted to provide optimal glycemic control. This is significant in intensive care units (ICUs), where it is crucial to administer insulin accurately to severely ill patients.

The adsorption of insulin differs markedly among various infusion materials. Research indicates that up to 57% of insulin is absorbed, thereby decreasing the quantity of insulin administered to the patient (1). Insulin adheres to plastic tubing, resulting in a reduced concentration of the insulin solution administered through an intravenous infusion set. The adsorption of insulin to PVC may result in insufficient glycemic control in critically ill patients, as reduced insulin availability in the bloodstream requires higher doses to attain the intended therapeutic effect (2, 3). This may lead to variability in blood glucose levels, thereby increasing the risk of hypoglycemia (4).

To make PVC flexible, it’s necessary to add plasticizers like Di Ethyl Hexyl Phthalate (DEHP). This can cause leaching into solutions and interaction with drugs, which could make them less effective and pose health hazards (5). Leaching may also affect how insulin is absorbed, since plasticizers can change the surface characteristics of PVC (6). PVC retains a considerable quantity of bacteriostatic additives from insulin preparations, potentially increasing the risk of infections if preservatives are not sufficiently administered (7).

The continued use of medical equipment containing PVC in critical care environments poses significant risks, particularly when in contact with insulin solutions. The research indicated that PVC catheters could retain up to 88% of the bacteriostatic preservatives in insulin formulations. This may diminish the efficacy of insulin in eradicating bacteria and increase the likelihood of skin infections in those undergoing continuous subcutaneous insulin infusion (CSII). This interaction underscores the necessity of meticulously evaluating catheter materials to guarantee patient safety and improve insulin therapy (7). Research indicates that the utilization of medical devices containing PVC in critical care environments poses significant risks, particularly for insulin adsorption (3, 8). This study revealed a significant reduction in insulin levels while utilizing PVC syringe extension lines (SEL), with concentrations decreasing to 30.1% after 60 min and 64.6% after 24 h. Adsorption can result in insufficient insulin delivery, hence jeopardizing glucose regulation in ICU patients.

In critical care settings, where precise glucose control is essential, the choice of infusion materials can significantly impact patient outcomes. Using PVC tubing may necessitate frequent adjustments to insulin dosage, potentially leading to fluctuations in blood glucose levels and an increased risk of adverse effects (3, 9). Insulin binds to PVC, which makes insulin delivery less effective. To get the same therapeutic effects, you need to use more insulin. This could make it harder to control blood sugar levels and raise the risk of hypoglycemia, especially in the first few hours of infusion when absorption is highest (10). Teaching healthcare workers on how infusion materials affect insulin delivery and encouraging the use of better materials can make patient care better and lower the risk of hypoglycemia (9).

Recent years have seen a growing awareness of the health and environmental issues linked to DEHP and PVC, leading to a transition toward alternative materials in the production of medical devices. DEHP-free and non-PVC infusion systems are increasingly utilized to reduce toxicological risks and contribute to sustainability initiatives in healthcare, commonly known as green hospital reforms. The potential advantages of these newer materials have not been thoroughly assessed regarding their effects on insulin stability and delivery. Evidence indicates that DEHP-free polymers may display varying adsorption characteristics, which raises concerns regarding their clinical equivalence and safety.

Green hospital reform is essential for mitigating PVC-related risks and advancing safer, more sustainable medical practices in critical care. The healthcare sector is a major source of pollution in the environment. By adopting green techniques, these effects can be lessened and patient safety and care quality can be improved (6, 11). Hospitals may benefit the environment and public health by reducing single-use plastics, optimizing resource use, and implementing sustainable waste management practices. This modification renders healthcare facilities less detrimental to the environment and aligns with broader public health objectives (12).

Hospitals produce significant amounts of plastic waste, predominantly consisting of single-use items, including PVC products. Minimizing these factors can lower the risk of nosocomial infections and exposure to endocrine disruptors, as well as mitigate environmental pollution. Replacing single-use plastics with sustainable alternatives can result in considerable economic and human resource savings, as evidenced by multiple case studies (13). To lessen the impact of healthcare institutions on the environment, it is important to put in place comprehensive waste management plans. This includes programs for recycling and training that teach people how to sort and throw away waste correctly (14). The implementation of the 5R concept (Reduce, Reuse, Recycle, Recover, and Rethink) in intensive care units can substantially decrease waste while maintaining care quality (15).

Green hospitals prioritize energy-efficient products that save money over time and are in line with green public procurement procedures, such as the Philips BlueSeal MRI (14). To provide practical strategies that enhance patient safety and environmental performance, healthcare sustainability science research and innovation are essential (11). For example, in the United States, the Occupational Safety and Health Administration (OSHA) reduced the VCM allowable exposure limit from 500 ppm to 1 ppm, which has helped reduce the occurrence of VCM-related health concerns (16).

Despite increasing awareness of this issue, there is still an absence of a unified understanding concerning insulin adsorption across various infusion systems, especially with contemporary materials and sustainability efforts. Considering the essential function of insulin in intensive care environments and the growing shift toward sustainable healthcare practices, a comprehensive understanding of the interaction between material science, pharmacokinetics, and clinical outcomes is vital. This narrative review seeks to summarize and critically evaluate the current literature on insulin adsorption to various intravenous infusion materials, contrasting typical PVC systems with DEHP-free choices. Moreover, it examines the ramifications of these discoveries for patient safety, glycemic regulation, device efficacy, and the shift toward sustainable healthcare systems.

Unlike prior reviews that have examined insulin adsorption, infusion material safety, or glycemic control independently, this review provides an integrated and multidisciplinary synthesis of these domains. It combines laboratory evidence, computational modeling, and available clinical data to evaluate material-dependent insulin adsorption within the context of critical care practice. Furthermore, this review uniquely links adsorption dynamics to patient safety implications and sustainable healthcare considerations, including DEHP exposure and green hospital procurement strategies. By bridging material science, clinical management, and environmental health perspectives, this article offers a comprehensive framework for evaluating infusion system selection beyond conventional discussions limited to device chemistry or glycemic protocols alone.

In light of these gaps, this narrative review synthesizes and critically appraises the current evidence on insulin adsorption in PVC and DEHP-free intravenous infusion systems, with emphasis on clinical safety, glycemic control, device performance, and sustainability considerations in critical care settings. Specifically, we compare material-dependent adsorption patterns, evaluate mitigation strategies used in clinical practice, and explore how material selection aligns with green hospital initiatives and environmentally responsible procurement policies.

## 2 Methods

This research employed a Structured Narrative Analysis Review (SNAR) design (17). This methodological approach facilitates a comprehensive synthesis and critical interpretation of the existing body of literature, drawing upon a wide range of scholarly sources. By adopting this approach, the review provides a detailed and nuanced perspective on the current landscape, offering both depth and breadth in evidence synthesis. Since the study designs, demographics, and outcomes measured were too dissimilar to be combined into a single number, a narrative method was selected. To ensure that the procedures were rigorous and transparent, however, systematic elements were incorporated, such as specific inclusion and exclusion criteria and a PRISMA flow diagram.

### 2.1 Search strategy

Searches were conducted systematically for literature published between 2000 and 2025 in PubMed, Scopus, Web of Science, CINAHL, ERIC, Medline, ScienceDirect, and Google Scholar, using a combination of Medical Subject Headings (MeSH) and free-text keywords. The following MeSH terms and subheadings were applied: “Insulin” [administration & dosage, pharmacology, therapeutic use], “Drug Compounding / Drug Stability” [adsorption, chemistry], “Infusions, Intravenous” [methods, adverse effects], “Equipment and Supplies” [polyvinyl chloride, medical device safety], “Diabetes Mellitus” [therapy, drug therapy, complications], “Critical Care” [nursing, methods, safety], and “Environment and Public Health” [sustainable development, green healthcare facilities].

The PICO (Population, Intervention, Comparison, Outcome) framework was applied to structure the search strategy and guide the review of the literature (18). The PICO tool ensured a focused and systematic approach to identifying relevant studies. This framework enhanced clarity, reproducibility, and rigor in the literature search process (Table 1). In addition, free-text keywords were incorporated to capture studies not indexed under MeSH, including “insulin adsorption,” “PVC infusion tubing,” “IV insulin safety,” “DEHP-free medical devices,” “critical care diabetes management,” and “green hospital initiatives.” Boolean operators (AND/OR) and truncation were used where appropriate to ensure comprehensive retrieval. The search strategy was adapted to fit the syntax of each database.

**Table 1:** PICO and MeSH search terms used in this review.

PICO element	Search terms	Category		
		MeSH headings	Subheadings	Keywords
Population (P)	Patients with diabetes mellitus receiving intravenous insulin therapy; healthcare professionals managing IV insulin in critical care settings	Diabetes Mellitus; Critical Care	Administration & dosage [ad]; Nursing [nu]	diabetes mellitus; critical care nurses
Intervention (I)	Use of PVC infusion tubing, DEHP-free medical devices, and intravenous insulin infusion protocols	Insulin; Infusions, Intravenous; Equipment and Supplies	Therapeutic use [tu]; Methods [mt]	insulin adsorption; PVC infusion tubing; DEHP-free medical devices; IV insulin protocols
Comparison (C)	Different types of infusion tubing (PVC vs. DEHP-free); alternative insulin delivery methods	Equipment and Supplies; Drug Stability	Adsorption [ad]; Chemistry [ch]	PVC vs. DEHP-free tubing; alternative insulin delivery
Outcome (O)	Insulin adsorption; infusion safety; glycemic control outcomes; device safety; sustainability in hospital practice	Drug Compounding; Environment and Public Health	Pharmacology [pd]; Adverse effects [ae]; Safety [sn]; Sustainable Development	insulin adsorption; infusion safety; glycemic control; device safety; green hospital initiatives

### 2.2 Inclusion and exclusion criteria

Inclusion and exclusion criteria were identified using the SPIDER (Sample, Phenomenon of Interest, Design, Evaluation, Research type) tool (Table 2). This was applied to guide the selection of studies for this review. This structured framework ensured a clear and systematic approach to defining inclusion and exclusion criteria, focusing on relevant populations, research designs, outcomes, and study types. By using SPIDER, the review process maintained transparency and minimized bias in study selection. Although quality assessment is not required for narrative reviews (19), a quality assessment of the research studies was undertaken using the Mixed Methods Appraisal Tool (MMAT) to enhance the overall rigor of the review process (20, 21).

**Table 2:** Inclusion and exclusion criteria (SPIDER tool).

SPIDER element	Inclusion criteria	Exclusion criteria
Sample (S)	Studies involving patients with diabetes mellitus receiving intravenous insulin therapy and healthcare professionals in critical care settings.	Studies not involving diabetic patients; studies with oral insulin or non-IV administration.
Phenomenon of Interest (PI)	Insulin adsorption in PVC or DEHP-free infusion tubing; safety and effectiveness of IV insulin delivery.	Studies unrelated to insulin adsorption or IV infusion systems.
Design (D)	Randomized controlled trials (RCTs), cohort studies, case–control studies, cross-sectional studies, and systematic reviews.	Editorials, opinion papers, narrative reviews, and conference abstracts without full data.
Evaluation (E)	Outcomes related to insulin stability, adsorption rate, device safety, glycemic control, and environmental sustainability.	Studies not reporting relevant outcomes (e.g., not measuring adsorption or insulin safety).
Research type (R)	Quantitative, qualitative, and mixed-method studies.	Animal studies are laboratory-only studies without clinical context.

A structured datasheet containing the data from the 38 research studies was meticulously created by us (Table 3). To ensure accuracy and minimize bias, the data abstraction process was carried out by two separate reviewers.

**Table 3:** Summary of studies datasheet (*n* = 38 studies).

No.	Author, year, and country	Title	Journal name	Objective	Study design	Setting and sample	Findings	Recommendations
1.	Jakobsson et al. (2009), Sweden and UK (10)	The impact of insulin adsorption onto the infusion sets in the adult intensive care unit	Journal of Diabetes Science and Technology	To explore insulin adsorption onto infusion sets in the laboratory and assess retrospectively if higher insulin doses are required after syringe changes in ICU practice.	Laboratory analysis + retrospective observational study	Laboratory test with PE tubing (Cardinal Health extension set, 200 cm, 1.6 mL) and 1 U/mL insulin; retrospective data from 109 ICU patients on a TGC protocol.	About 10% insulin adsorption occurred in the first hour at 1 mL/h infusion rate; no significant adsorption at 4 mL/h. In ICU patients, insulin infusion rate had to be increased after set change when infusion ≤ 1 mL/h (76.5% of cases).	At low infusion rates (≤1 mL/h), priming is important; awareness is needed that only ~90% of expected insulin may be delivered during the first 3 h after set change.
2.	Fuloria et al. (1998), USA (32)	Effect of flow rate and insulin priming on the recovery of insulin from microbore infusion tubing	Pediatrics	To examine the effect of flow rate and insulin priming on insulin recovery from PVC and PE-lined infusion tubing in extremely low birth weight (ELBW) infants.	In vitro experimental study	Laboratory study using microbore PVC tubing and PE-lined PVC tubing; insulin concentrations tested at flow rates 0.05 and 0.2 mL/h.	Significant insulin loss occurred with unprimed PVC tubing at low flow rates (as low as 11–27% recovery in early hours). Priming with 5 U/mL insulin for 20 min markedly improved recovery (~70% early, near 100% by 8 h). PE-lined tubing showed reduced loss compared to plain PVC, especially at higher flow rates.	Prime tubing with higher concentration insulin before clinical use to saturate binding sites and enhance recovery. Prefer PE-lined tubing over plain PVC for improved insulin delivery accuracy.
3.	Hirsch et al. (1981), USA (27)	Insulin adsorption to polyolefin infusion bottles and polyvinyl chloride administration sets	American Journal of Hospital Pharmacy	To evaluate insulin adsorption to polyolefin infusion bottles when used with conventional PVC administration sets.	In vitro experimental study using ^125I-labeled insulin	Laboratory setting; IV solutions containing 20, 40, 70, and 100 units of insulin were infused through polyolefin bottles with PVC administration sets at 125 mL/h for up to 7 h.	Greater adsorption occurred at lower insulin concentrations. Saturation limits were identified as 7.8 units for the system overall, 2.7 units for the PVC administration set, and 5.4 units for the polyolefin bottle. Adsorption from polyolefin bottles was lower compared to PVC bags reported in other studies.	Insulin adsorption is concentration-dependent; using higher initial concentrations and considering material type (polyolefin vs. PVC) can reduce insulin loss in clinical practice.
4.	Tokhadze et al. (2019), France (6)	Impact of alternative materials to plasticized PVC infusion tubings on drug sorption and plasticizer release	Scientific Reports	To investigate the effect of alternative tubing materials (PVC coextruded with PE, PU, SEBS; monolayer SEBS and TPO) compared to plasticized PVC on drug sorption (insulin, diazepam, paracetamol) and plasticizer migration.	In vitro experimental study (static and dynamic infusion simulations at 1 mL/h and 10 mL/h)	Laboratory tests with six tubing types; three drugs (insulin, diazepam, paracetamol) infused and analyzed by HPLC, ATR-FTIR, zeta potential, and GC–Ms	In that study, sorption patterns varied by drug and tubing composition. PVC and PVC/PU showed the greatest sorption of diazepam, while insulin adsorption differed across material architectures and was not consistently reduced by DEHP-free alternatives. Overall, adsorption behavior remained dependent on both material composition and device design, and some alternative polymers (e.g., SEBS, TPO, PVC/PE) reduced diazepam sorption and plasticizer release compared with conventional PVC	SEBS, TPO, and PVC/PE appear as safer alternatives to PVC, minimizing plasticizer migration and sorption losses. However, insulin adsorption remains a challenge, highlighting the need for further optimization of tubing material choice based on the infused drug.
5.	Knopp et al. (2019), New Zealand (50)	Modeling insulin adsorption in intravenous infusion sets in the intensive care unit	IFAC Journal of Systems and Control	To develop and validate a mathematical model describing insulin adsorption in infusion sets, accounting for tubing material, flow rate, and other variables, in order to improve glycaemic control in ICU patients.	Modeling study using a two-compartment approach (free vs. bound insulin), validated with published experimental data	In silico modeling based on prior experimental studies of insulin adsorption in PVC and PE infusion tubing under ICU-relevant flow rates and concentrations	The two-compartment model accurately reproduced experimental adsorption dynamics, showing substantial insulin losses (20–60%) at low flow rates, particularly in neonatal settings. Parameter variability across studies highlighted material- and flow-dependent effect	Incorporating insulin adsorption dynamics into clinical glycaemic control protocols is essential to avoid underdosing and poor glucose regulation. Further experimental studies with standardized methods are needed to refine and expand model applicability.
6.	Massé et al. (2018), France (28)	In vitro assessment of the influence of intravenous extension set materials on insulin aspart drug delivery	PLOS ONE	To evaluate the effect of different intravenous extension set materials (PVC, PE, and PE/PVC) on the delivery of insulin aspart, human insulin, and their preservatives (phenol, metacresol).	In vitro experimental laboratory study using UFLC-DAD for quantification.	Laboratory setting; infusion of Novorapid (insulin aspart) and Umuline rapide (human insulin) at 1 IU/mL, delivered at 2 mL/h over 24 h through 16 extension lines (8 PVC, 3 PE, 5 PE/PVC).	Early adsorption observed: human insulin loss was highest with PVC (~24% at 30 min), minimal with PE (~3%), and moderate with PE/PVC (~19%). Insulin aspart showed smaller initial loss (~5–11%) across all materials. Over 24 h, insulin recovery approached ~100% with all materials. Preservatives (especially metacresol) were significantly adsorbed by PVC (up to ~50% loss), while recovery with PE and PE/PVC remained close to theoretical values.	Prefer PE or PE/PVC extension sets for continuous insulin infusion to minimize preservative adsorption and maintain insulin stability. Consider clinical evaluation of preservative loss implications, as they play a key role in maintaining insulin conformation and safety.
7.	Gonzales et al. (2025), USA/India (30)	Understanding the sorption of paraben preservatives and implications for insulin stability in PVC infusion sets	Journal of Biomedical Materials Research	To investigate sorption mechanisms of methylparaben and propylparaben on PVC and FEP tubing surfaces using molecular dynamics simulations and experiments, with implications for insulin stability.	Computational molecular dynamics simulations with experimental validation	PVC and FEP tubing materials; simulations and sorption experiments with methylparaben and propylparaben	- Both parabens undergo significant sorption to PVC but minimal to FEP.	To minimize preservative loss and maintain insulin stability, avoid the use of PVC infusion tubing for insulin preparations containing parabens. Fluorinated ethylene propylene (FEP) tubing should be preferred, as it demonstrated minimal sorption of methylparaben and propylparaben, thereby preserving insulin integrity during infusion.
8.	Seifi etal (2004), Iran (8)	Insulin adsorbance to polyvinylchloride (PVC) surfaces of fluid container and infusion-set	Middle East Journal of Anaesthesiology, 17(5), 975–981	To clarify the binding sites of insulin on PVC surfaces and propose solutions to reduce insulin loss	Experimental laboratory study	Four 1,000 mL PVC bottles containing 5% dextrose with added insulin; infusion at 100 mL/h; samples collected at 0, 15, 30, 45, and 60 min	Insulin concentration in PVC bottles remained stable, but insulin levels at infusion set terminal significantly decreased over 60 min (*p* = 0.004), confirming adsorption at infusion set surfaces	Increasing the initial insulin dose and using alternative infusion sets (e.g., polyethylene) may reduce insulin loss and improve delivery accuracy
9.	Zahid et al. (2008), UK (25)	Adsorption of insulin onto infusion sets used in adult intensive care unit and neonatal care settings	Diabetes Research and Clinical Practice	To investigate insulin adsorption on infusion sets used in adult ICU and neonatal care, evaluating the impact of tubing material, dimensions, and flow rate.	Experimental laboratory study with continuous flow UV analysis	Infusion tubing used in adult ICU and neonatal settings; insulin concentration monitored under flow conditions	- Insulin adsorption varies with tubing composition, dimensions, and flow rate.	Insulin adsorption should be carefully considered when selecting infusion sets for ICU and NICU patients. Clinicians should avoid using long or narrow-bore PVC tubing at low flow rates, and whenever possible, employ alternative materials or preconditioning strategies to minimize early insulin loss.
10.	Bernard et al. (2023), France (ARMED study group) (37)	Medical devices used in NICU: The main source of plasticisers’ exposure of newborns	Science of the Total Environment	To assess the exposure of newborns in NICU to plasticisers leaching from medical devices, and compare urinary metabolites of DEHP, DEHTP, and TEHTM.	Multicentric biomonitoring study	97 NICU patients; 508 urinary samples collected daily during NICU stay and at discharge	- Newborns exposed mainly to DEHP metabolites, levels 5–10 times higher than DEHTP and up to 228 times higher than TEHTM.	In neonatal intensive care, the use of PVC/DEHP-containing medical devices should be minimized or replaced by safer alternatives. Hospitals should prioritize PVC-free and DEHP-free infusion systems to reduce toxic plasticiser exposure in newborns, a population highly vulnerable to endocrine and developmental risks.
11.	Knopp et al. (2021), New Zealand (9)	Clinical Recommendations for Managing the Impact of Insulin Adsorptive Loss in Hospital and Diabetes Care	Journal of Diabetes Science and Technology	To provide clinical recommendations for managing insulin adsorptive loss in infusion sets across ICU and outpatient contexts, using dynamic adsorption modeling and review of preconditioning methods.	Dynamic adsorption model with clinical review	Outpatient pump therapy (children and adults); ICU patients (adult, pediatric, neonatal)	- Up to 80% of insulin lost in first hour of outpatient pump therapy; losses become negligible thereafter.	Clinical glycaemic control protocols should explicitly account for insulin adsorptive losses in infusion systems, particularly during the initial hours of therapy and at low flow rates. Preconditioning or priming of tubing should be incorporated into standard practice, and infusion materials should be selected to minimize adsorption, especially in neonatal and critical care settings.
12.	Aldhaeefi et al. (2022), Saudi Arabia (51)	Updates in the Management of Hyperglycemic Crisis	Frontiers in Clinical Diabetes and Healthcare, 2, 820,728	To review DKA and HHS management based on recent evidence and suggest practical pathways	Narrative clinical review	Evidence synthesis; no direct patient data	DKA mortality <1%, HHS ~ 15%; insulin loss in PVC tubing; complications include electrolyte imbalance and cerebral edema	When managing diabetic ketoacidosis (DKA) and hyperosmolar hyperglycemic state (HHS), clinicians should use continuous IV insulin infusion without bolus dosing and always flush PVC infusion tubing with 20 mL solution to minimize adsorption-related insulin loss and ensure accurate delivery. Careful monitoring is essential during transition to subcutaneous insulin.
13.	Alkaabi and Aljaradin (2023), United Arab Emirates (40)	Green Hospitals for the Future of Healthcare: A Review	Al-Kitab Journal for Pure Sciences, 6(2), 31–45	To review the green hospital concept and its role in sustainable healthcare	Narrative review	Conceptual analysis; UAE and international examples	Green hospitals improve human and environmental health; barriers include cost and limited policies	Government legislation and incentives; hospital sustainability committees; eco-friendly procurement and innovative design
14	Mian et al. (2022), Netherland (35)	Adsorption of insulin onto neonatal infusion sets: should intravenous administration of insulin to treat hyperglycemia in preterm babies on the NICU be proceeded by priming of the intravenous system, adding of albumin, or non-priming to get to a stable insulin dose?	Molecular and Cellular Pediatrics, 9(1), 20	To determine whether priming, preconditioning, adding albumin, or non-priming yields more stable IV insulin dosing in preterm neonates.	Evidence-based brief review of in-vitro studies (no in-vivo neonatal trials found).	Literature synthesis; 8 relevant in-vitro studies of neonatal infusion lines.	Priming improves insulin recovery vs. non-priming; preconditioning + priming provides the smoothest early delivery; albumin can increase recovery but raises safety concerns; adsorption is highest at start, greater at low flow rates and with PVC vs. polyolefins; stable dose typically after the first 1–6 h.	Prefer preconditioning + priming before neonatal IV insulin; if not feasible, at least prime (e.g., ~20 mL per some studies); avoid albumin unless justified; closely monitor glucose in first hours due to early losses and rebound.
15	Braun Medical Inc. (2025), USA (38)	Products not made with Diethylhexyl Phthalate (DEHP) and Polyvinyl Chloride (PVC)	B. Braun USA official website (Patient and Provider Safety)	To inform healthcare providers about the risks of DEHP/PVC exposure and present B. Braun’s safer alternatives.	Industry/gray literature (corporate safety document)	Informational data; no clinical sample	DEHP may account for up to 40% of the weight of PVC IV bags and tubing. Patient exposure to DEHP is linked to carcinogenic, reproductive, and developmental risks. High-risk groups include neonates, pediatric patients (especially males), pregnant/lactating women, and oncology patients. Approximately 70–75% of U. S. IV/irrigation containers still contain PVC/DEHP. Professional bodies (e.g., AMA) have called for the phase-out of DEHP/PVC medical devices.	Healthcare providers should transition to DEHP-free and PVC-free infusion products (e.g., CARESAFE™ IV sets), especially for high-risk populations such as neonates, pediatric patients, pregnant/lactating women, and oncology patients. Hospitals and regulators should update procurement policies to prioritize safer alternatives and reduce toxic plasticiser exposure
16	El Safty (2025), Egypt (41)	The Concept of Green Hospitals and Sustainable Practices	Egyptian Journal of Occupational Medicine, 49(1), 117–123	To review the research landscape of green hospitals and highlight sustainable practices within the healthcare sector.	Narrative review article	Conceptual review; not a primary sample	Healthcare emits pollutants green hospitals use renewable energy smart systems and waste reduction benefits include better outcomes and cost-effectiveness barriers are certification and policy gaps	Adopt UN SDGs implement renewable energy pursue certification engage community and prioritize sustainability in leadership
17	Hardy et al. (2018), New Zealand (52)	Model based insulin absorption into intravenous infusion sets in adult and neonatal intensive care unit’s	IFAC-PapersOnLine, 51(27), 105–110	To develop and validate a mathematical compartment model to describe insulin absorption into infusion tubing materials (PVC and PE) in adult and neonatal ICU settings.	Modeling study based on literature data, using conservation of mass equations and Matlab fitting to estimate insulin absorption (K1) and release (K2) parameters.	Literature-derived data on insulin infusions through PVC and PE tubing; model validation against experimental data sets.	The model fit experimental data well, with a max 8.54% difference. Insulin absorption was more variable in PVC than in PE tubing. Identified absorption (K1) and release (K2) rates were of similar magnitude, but more inconsistent in PVC. Variability attributed to tubing material and assumptions about insulin concentration in syringes.	Mathematical modeling can reliably predict insulin loss due to tubing adsorption. Explicit validation with standardized experiments is needed. PVC tubing shows less predictable insulin delivery compared to PE, supporting the use of PE in clinical practice.
18	European Commission, SCHEER (2024), European Union (53)	Update of the guidelines on the benefit–risk assessment of the presence of phthalates in certain medical devices	Publications Office of the European Union	To provide updated EU guidance on benefit–risk assessment of CMR/ED phthalates in medical devices.	Expert Committee Guideline (Regulatory Report)	Not applicable (regulatory update and expert consultation)	Phthalates (including DEHP) in medical devices remain a safety concern, particularly due to their carcinogenic, mutagenic, and reprotoxic (CMR) and endocrine-disrupting (ED) properties. Despite regulatory changes, data gaps persist regarding toxicity levels and patient exposure. Clinical benefit–risk assessments (BRA) are required when phthalates are present, but the quality and transparency of submitted data vary. Safer alternatives to DEHP are increasingly available, but comparative safety data are still limited.	Substitute DEHP-containing PVC devices with safer alternatives whenever possible. Require robust, high-quality toxicological and exposure data in BRA submissions. Improve transparency in regulatory reporting. Encourage generation of new clinical and toxicological evidence to guide safer substitution.
19.	Fayon et al. (2025), France (48)	Interactions of insulin aspart hexamer and excipients with plasticized polyvinyl chloride surfaces: A comprehensive investigation combining molecular simulations and experiments	International Journal of Biological Macromolecules, 319(Pt 2), 145,043	To characterize the adsorption and absorption of insulin aspart hexamer and phenolic excipients on plasticized PVC surfaces.	Experimental study + Molecular dynamics simulations	Laboratory-based experiments; molecular simulation models; insulin aspart formulations	Insulin hexamer adsorbs almost instantaneously to PVC-rich regions. Phenolic excipients (phenol, metacresol) absorbed into plasticized PVC. Thermodynamic modeling confirmed adsorption energetics and diffusion behavior.	Advance coarse-grained modeling for PVC phase heterogeneity. Predict biomacromolecule interactions in medical infusion systems. Develop safer non-PVC alternatives for clinical infusion.
20.	Møllmann et al. (2006), Denmark/Netherlands/Sweden (54)	Interfacial adsorption of insulin: Conformational changes and reversibility of adsorption	European Journal of Pharmaceutical Sciences, 27(2–3), 194–204	To investigate adsorption of human insulin to hydrophobic surfaces, focusing on conformational changes and reversibility of adsorption.	Comparative experimental study (TIRF, circular dichroism, fluorescence spectroscopy)	Human insulin adsorbed on Teflon particles; structural analyses	High-affinity adsorption observed even under repulsive electrostatic conditions. Adsorbed insulin showed unfolding with loss of α-helix and increase in random coil. Adsorbed insulin displayed altered thermal stability compared to solution state. Reversibility of adsorption was unclear; desorption mechanisms differed between labeled vs. unlabeled insulin.	Adsorption leads to structural destabilization; must be considered in insulin formulations and device-material design. Future studies should evaluate long-term reversibility and stability impacts.
21.	Newby and Holmes (2017), Canada (34)	Effect of Tubing Flush or Preconditioning on Available Insulin Concentration for IV Infusion: A Pilot Project	Canadian Journal of Hospital Pharmacy, 70(4), 320–321	To compare the effect of flushing vs. preconditioning of polyethylene-lined tubing on available insulin concentration during IV infusion.	Pilot experimental study	Two 500 mL bags of insulin solution (0.1 U/mL) infused via polyethylene-lined tubing; insulin concentrations measured at baseline and after infusion	Both flushing and preconditioning led to reduced available insulin compared to initial concentration. Preconditioning resulted in more consistent delivery (73.8% at start, 68.5% after 1 h). Flushing resulted in very low delivery (21% after 1 h).	Preconditioning of polyethylene-lined tubing should be considered to achieve more consistent insulin delivery. Larger studies needed to optimize preconditioning time and evaluate 24 h delivery.
22.	Practice Greenhealth (2025), USA (42)	Safer medical products and devices	Practice Greenhealth (online resource)	To provide hospitals with strategies and policies to eliminate PVC and DEHP from clinical devices and products.	Policy guidance and institutional case examples	Hospitals and health systems adopting safer chemicals policies; NICU and other high-risk product categories	Many hospitals adopted PVC- and DEHP-free purchasing policies. FDA cautioned against DEHP exposure in neonates/infants. Priority product categories identified (e.g., infusion sets, catheters, enteral tubes).	Eliminate PVC/DEHP from at least 2 high-priority product categories. Implement step-by-step substitution programs and measure outcomes.
23	Robert et al. (2021), France (31)	Impact of insulin adsorption in various containers during hyperkalaemia treatment	Clinical Kidney Journal, 14(10), 2,255–2,260	To assess variability in effective delivered insulin during hyperkalaemia treatment using different containers (bags, syringes).	Experimental in vitro study with chromatographic assays (HPLC)	Insulin-glucose infusions prepared in PE, glass, PVC bags and syringes; effective insulin delivery measured	PE bag showed lowest delivery (63.3% of expected). PVC bag retained highest delivery (93.8%). Syringe procedures reached ~90% with no major variation. Delivery influenced by insulin concentration and procedure duration.	Use polypropylene syringes to minimize insulin delivery variation. Clinical hyperkalaemia protocols should specify container type. Future clinical studies should detail infusion procedures precisely.
24	Goldberg et al. (2006), USA (33)	“Waste Not, Want Not”: Determining the Optimal Priming Volume for Intravenous Insulin Infusions	Diabetes Technology and Therapeutics	To determine the optimal priming volume required to minimize insulin adsorption losses in IV infusion lines.	Experimental laboratory study (brief report).	20 insulin infusion bags (100 U regular insulin in 100 mL NaCl); polypropylene infusion set; effluent collected at 10 mL intervals up to 50 mL.	Without priming, insulin concentration was 15.8% lower than maximal; 10 mL prime reduced loss to 6.6%; 20 mL prime eliminated significant adsorption loss (3.4%). Priming >20 mL showed no additional benefit.	Prime IV insulin infusions with 20 mL to reduce insulin adsorption and avoid unnecessary waste; larger priming volumes increase cost and workload without clinical benefit.
25.	Scientific Committee on Emerging and Newly Identified Health Risks (2015), European Union (62)	Opinion on the safety of medical devices containing DEHP-plasticized PVC or other plasticizers on neonates and other groups possibly at risk (2015 update)	European Commission, Scientific Committee on Emerging and Newly-Identified Health Risks (SCENIHR)	To assess updated evidence on DEHP exposure from medical devices and potential health risks in high-risk groups (neonates, dialysis patients, ECMO, etc.).	Expert scientific opinion; systematic review of published toxicological, clinical, and epidemiological studies (2008–2015).	Focus on medical device use in neonates, children, adults with chronic conditions (dialysis, transfusion, ECMO).	DEHP exposure from medical devices can exceed TDI (50 μg/kg bw/d) many-fold in NICU neonates (up to 6,000 μg/kg bw/d) and in dialysis patients (up to 2,200 μg/kg bw/d). Risks include reproductive and developmental toxicity; high concern for neonates and ECMO patients. Alternatives exist but data on their leaching and toxicity are limited.	Whenever possible, replace DEHP-containing PVC medical devices with safer alternatives, particularly in high-risk groups (neonates, dialysis, ECMO). Where substitution is not yet feasible, perform a robust benefit–risk assessment (BRA) and generate stronger toxicological data on alternative plasticizers to ensure patient safety.
26.	Sürmelioğlu et al. (2021), Turkey (1)	Evaluation of regular insulin adsorption to polypropylene bag and polyvinyl chloride infusion set	International Journal of Clinical Practice, 75(4), e13895	To evaluate insulin adsorption rates in PP bags and PVC infusion sets and assess insulin stability during infusion.	Experimental in vitro study using HPLC analysis	100 IU regular insulin in PP bags with NaCl solution (n = 6); infusion at 2 mL/h using PVC sets; storage at +25 °C vs. + 4 °C	Insulin adsorption to PVC sets reached 57% at 24 h. Adsorption to PP bags was ≤5%. No significant stability differences between storage at +25 °C and +4 °C.	Use PP bags for insulin infusion; 24-h change acceptable. Losses from PVC sets are clinically significant, avoid if possible.
27.	Thompson et al. (2012), USA (55)	The effect of tubing dwell time on insulin adsorption during intravenous insulin infusions	Diabetes Technology and Therapeutics, 14(10), 912–916. https://doi.org/10.1089/dia.2012.0098	To determine the effect of tubing dwell time on insulin concentration from intravenous infusion sets.	In vitro experimental study.	Insulin solutions (0.1, 1, 10 U/mL) dwelled in polypropylene IV infusion sets for 0, 15, 30, or 60 min; each tested in quintuplicate.	Mean insulin concentrations from tubing were not significantly different between dwell times. Dwell time did not affect insulin adsorption in polypropylene tubing.	Recommend starting insulin infusions without dwell time after a 20-mL flush; this avoids delays and improves efficiency of infusion preparation.
28.	Tokhadzé et al. (2022), France (29)	Insulin Adsorption onto PE and PVC Tubings	ACS Applied Bio Materials, 5(6), 2,567–2,575. https://doi.org/10.1021/acsabm.2c00029	To compare insulin adsorption dynamics on polyethylene (PE) versus PVC tubing through experimental (HPLC) and computational simulation approaches.	Combined in vitro HPLC measurements and molecular simulations.	Tubings made of PE and plasticized PVC; insulin adsorption quantified experimentally and modeled via Gibbs free energy profiles.	Strong agreement between simulated free energy of adsorption and HPLC data. Adsorption accompanied by conformational changes in insulin and specific interfacial arrangements of polymer, water, insulin, and plasticizer molecules.	Findings highlight material-dependent insulin adsorption. Results support selection of tubing materials (e.g., PE over PVC) to minimize insulin loss in infusion systems.
29.	Van Vliet et al. (2011), USA (39)	A review of alternatives to di(2-ethylhexyl) phthalate-containing medical devices in the neonatal intensive care unit	Journal of Perinatology, 31(8), 551–560. https://doi.org/10.1038/jp.2010.208	To review substitute compounds and alternative medical products to replace PVC/DEHP in NICU medical devices and conduct an inventory analysis.	Systematic literature review + NICU product audit.	Audit of a large metropolitan NICU (2005–2006); 21 products assessed for DEHP content; availability of alternatives checked via databases, company websites, and interviews.	Two approaches identified: (1) DEHP-free plasticizers; (2) replacing PVC entirely. Nearly 48% of products were DEHP-free. PVC-free polymers seen as safer substitutes but significant toxicological data gaps remain.	Support transition to PVC-free polymers in NICUs; call for systematic toxicological testing and continued development of safer alternatives.
30.	Mansuri et al. (2025), India (56)	Phthalate Exposure: Prevalence, Health Effects, Regulatory Frameworks, and Remediation	Chemical Research in Toxicology, 38(8), 1,291–1,308	Review prevalence, health risks, and regulations of phthalate exposure with focus on mitigation	Narrative review	Literature-based analysis, not primary subjects	Phthalates widely present, linked to endocrine disruption, oxidative stress, reproductive and developmental toxicity, with evidence of bioaccumulation and regulatory gaps	Strengthen regulatory frameworks, improve monitoring methods, and implement remediation strategies to reduce environmental and health risks
31.	Sahnoune Millot et al. (2025), France (57)	Coarse-grained insights into insulin aspart adsorption on PVC surfaces	The Journal of Physical Chemistry B, 129(27), 6,997–7,009	To investigate insulin aspart adsorption on PVC infusion surfaces using molecular dynamics simulations combined with experimental validation	Computational and experimental analysis	Simulations (Martini 3 coarse-grained) + lab validation on PVC materials	Insulin aspart strongly adsorbs onto PVC matrix rather than plasticizers Adsorption occurs for both monomeric and hexameric forms Explains clinically observed insulin loss in PVC infusion systems	Prefer non-PVC/DEHP-free tubing in clinical infusion Incorporate molecular-level evidence into guidelines and procurement policies
32.	American Diabetes Association Professional Practice Committee (2025), USA (43)	Diabetes Care in the Hospital: Standards of Care in Diabetes-2025	Diabetes Care, 48(Suppl. 1), S321–S334	o provide updated evidence-based standards for diabetes care in hospitalized patients	Clinical practice guideline (expert committee consensus)	Not a primary study; guideline for hospital-based diabetes management	Defined key standards for inpatient diabetes care, including insulin use, glucose monitoring, and quality metrics.	Implement evidence-based hospital protocols; train healthcare teams; use continuous monitoring and standardized pathways to improve safety and outcomes.
33.	Desgrouas et al. (2023), France (58)	Insulin therapy and blood glucose management in critically ill patients: A 1-day cross-sectional observational study in 69 French intensive care units	Annals of Intensive Care, 13(1), 53	To describe insulin use practices and glycemic control management in French ICUs	Multicenter 1-day cross-sectional observational study	69 ICUs in France; 893 adult patients	Marked variability in insulin protocols and blood glucose targets 45% of patients experienced hyperglycemia; 2.9% experienced hypoglycemia Subcutaneous insulin use was linked to higher hyperglycemia incidence IV insulin protocols often failed to prevent hyperglycemic events	Standardize insulin infusion protocols across ICUs Prefer IV insulin with strict monitoring to minimize hyperglycemia Reduce reliance on SC insulin in critical care settings
34.	Dhatariya and Umpierrez (2024), USA (59)	Management of diabetes and hyperglycemia in hospitalized patients	Endotext (NCBI Bookshelf)	Summarize current evidence and ADA 2025 recommendations for managing diabetes and hyperglycemia in hospitalized patients	Narrative clinical review / guideline update	Hospitalized adult patients (ICU and non-ICU)	Hyperglycemia common in 22–46% of inpatients Associated with complications and mortality IV insulin is preferred in ICU; subcutaneous insulin in non-ICU New ADA 2025 updates include cautious use of SGLT2 inhibitors and possible use of DPP4 inhibitors with basal insulin	Maintain glycemic targets: 140–180 mg/dL (ICU), 100–180 mg/dL (non-ICU) Use IV insulin in critically ill patients Individualize goals if achievable without hypoglycemia Avoid GLP-1 agonists in hospital setting
35.	El-Kebbi et al. (2021), MENA Region (60)	Epidemiology of type 2 diabetes in the Middle East and North Africa: Challenges and call for action	World Journal of Diabetes, 12(9), 1,401–1,425	To review the prevalence, risk factors, and challenges of type 2 diabetes in MENA	Narrative review	20 MENA countries, epidemiological data	MENA has the world’s highest diabetes prevalence (12.2% in 2019) with early onset in youth Main drivers: obesity, inactivity, urbanization, poor nutrition, and high consanguinity Large gaps exist in data accuracy, funding, and healthcare access	strengthen national strategies for diabetes prevention and control Promote lifestyle modification and education programs Address socioeconomic disparities and gender-related risks Improve screening, data collection, and healthcare system response
36.	Hewson et al. (2001), Australia (36)	Insulin infusions in the neonatal unit: Delivery variation due to adsorption	Journal of Paediatrics and Child Health	To assess variability in insulin delivery due to adsorption under neonatal unit conditions, examining diluent, preconditioning, flushing, concentration, flow rate, catheter type, and albumin addition.	Experimental (simulated insulin infusions, *in vitro*)	Laboratory simulation of neonatal insulin infusions over 22 h.	Significant insulin loss at low concentrations/flow rates. Preconditioning + flushing improved stability. Albumin addition also reduced adsorption.	Use preconditioning/flushing or albumin addition to ensure consistent insulin delivery in neonatal infusions.
37.	Peterson et al. (1976), USA (26)	Insulin adsorbance to polyvinylchloride surfaces with implications for constant-infusion therapy	Diabetes	To test insulin adsorption in PVC infusion sets and assess need for additives like albumin	In vitro experimental study with radiolabeled and unlabeled insulin	PVC infusion bags and sets; insulin in saline or Ringer’s	Up to 70% insulin loss in early effluent; recovery >90% after washout; albumin prevented loss but not essential	Use ≥25 U/500 mL insulin and wash out 50 mL before infusion; no need for albumin if priming applied
38.	Hirsch et al. (1977), USA (61)	Clinical significance of insulin adsorption by polyvinyl chloride infusion systems	American Journal of Hospital Pharmacy	To determine clinical relevance of insulin adsorption in PVC infusion systems	Laboratory and clinical assessment	PVC infusion sets in hospital setting	Significant insulin loss at low concentrations; adsorption clinically relevant	Material choice critical; higher insulin concentrations reduce adsorption

Using Braun and Clarke’s six-phase methodology, a thematic analysis was carried out: (1) familiarizing data; (2) creating preliminary codes; (3) forming themes; (4) reviewing themes; (5) defining and labeling themes; and (6) using example data to illustrate findings (22). This approach enabled the identification of recurring patterns across various studies while also acknowledging variations in methodology and context. The JBI Manual for Evidence Synthesis’s best practice guidelines for evidence synthesis also served as a reference for the review process (23). This handbook provided a framework for ensuring that narrative reviews were transparent, consistent, and methodologically sound.

## 3 Results

**Figure 1:**
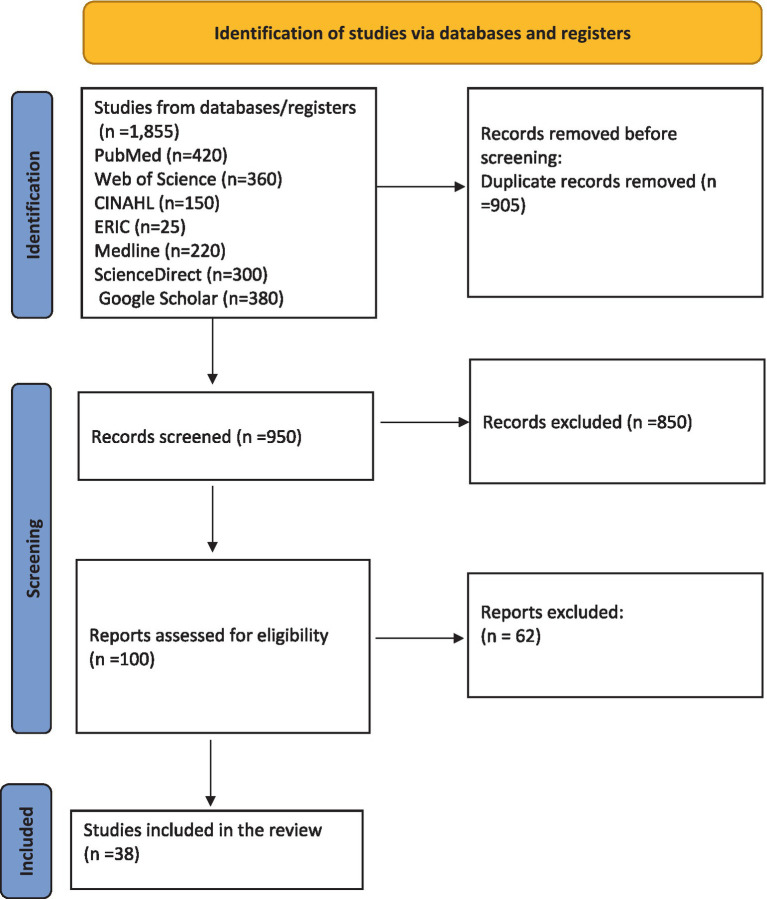
PRISMA flow diagram of the literature review process.

A total of 1855 papers were first identified via database searches. Following the elimination of duplicates, 950 distinct records were retained for evaluation. All publications were independently evaluated by two members of the study team (RI and AH) utilizing the Covidence literature screening software.^1^ According to the exclusion criteria, 850 papers were deemed unrelated to the study’s purpose. A total of 45 full-text papers were evaluated for eligibility. One hundred relevant articles were subjected to full-text screening, of which 38 were deemed appropriate for inclusion in the review. Figure 1 is a Preferred Reporting Items for Systematic Reviews and Meta-Analyses (PRISMA) flowchart that encapsulates the search strategy (24).

Two authors (RKI and AH) extracted and structured the material into an electronic and paper datasheet (Table 3) to ease the review and interpretation of findings. The datasheet was organized into eight sections: author(s)/year/country, title, journal name, purpose of study, study design, setting/sample, findings, and suggestions.

This review identified 38 articles, with 11 conducted in the United States, eight in France, three in New Zealand, two each in India and the European Union, and one study each in the United Kingdom, Sweden, Iran, Saudi Arabia, the United Arab Emirates, Egypt, the Netherlands, Denmark, Turkey, the MENA region, and Australia.

The predominant type of studies was experimental laboratory investigations (n = 15, 39%), followed by computational and mathematical modeling studies (n = 6, 16%). Two studies (5%) employed a pilot or small-scale experimental design, whereas two studies (5%) were multicenter observational clinical investigations. One study (3%) employed a combined laboratory and retrospective clinical methodology, while another study (3%) constituted an evidence-based, succinct assessment of *in vitro* data. Furthermore, 11 studies (29%) comprised narrative reviews, therapeutic guidelines, or policy documents.

Thematic analysis employs a methodology consisting of six interconnected phases that form the cyclic framework. Initially, relevant data from the chosen studies were collected, encompassing study objectives, design, setting, and results related to insulin adsorption and infusion materials. By analyzing the collected data, researchers obtained insights concerning the evidence associated with intravenous insulin administration and the safety of the devices. Further open coding was employed to discern themes, including adsorption rates, tube materials, infusion protocols, and sustainability issues. Descriptive codes were employed to encapsulate the essential contributions of each study, which were subsequently organized into main themes. Codes were discerned and classified into four principal themes through the examination of their patterns, interconnections, and resemblances: (1) Insulin Adsorption and Clinical Safety; (2) Material-Dependent Variability; (3) Mitigation Strategies in Practice; (4) Green Hospital and Sustainability Implications.

### 3.1 Insulin adsorption and clinical safety

The findings from the reviewed research indicate that insulin adsorption presents a significant clinical safety issue. PVC infusion sets absorb a lot of insulin, especially when the flow rate is low and at the beginning of the infusion. Documented losses of up to 70% can result in underdosing, which is clinically relevant and may compromise glycemic control. This issue is especially dangerous in critical care and newborn settings, when careful glucose control is essential and even little changes in insulin supply might raise the risk of hypoglycemia and hyperglycemia. Clinical studies indicate that insulin infusion rates frequently require augmentation following set alterations, underscoring that adsorption constitutes a genuine patient safety concern rather than only a laboratory observation (8, 10, 25–27).

### 3.2 Material-dependent variability

The adsorption of insulin varied significantly based on the infusion medium utilized. PVC consistently showed the most insulin loss, while other materials like polyethylene (PE), PE/PVC blends, fluorinated ethylene propylene (FEP), and polypropylene showed less insulin loss. For instance, studies showed that using PE tubing almost completely recovered insulin, but using PVC configurations caused a lot of early losses. There is still significant variation across alternatives, though. For example, SEBS and TPO reduced plasticizer leaching but did not entirely stop insulin adsorption. These studies underscore that the selection of materials significantly influences insulin stability, and not all options are equally effective in minimizing adsorption (1, 6, 28–31).

### 3.3 Mitigation strategies in practice

Researchers have looked into and found that a number of ways to lower insulin absorption in clinical practice are effective. One of the most reliable results is that priming or preconditioning infusion devices with insulin before they are used in a clinical setting greatly reduces initial losses. Some studies have shown that priming with insulin at a higher concentration or flushing with at least 20 mL of solution can bring insulin recovery back to almost 100%. Changing the flow rates also helps to lower adsorption; higher infusion rates lower early binding. Although albumin supplementation was sometimes helpful, it was not safe for babies. Clinical guidelines stress the importance of regular priming, flushing, and careful choice of tube materials to ensure accurate insulin delivery and long-term glucose management (9, 32–36).

### 3.4 Green hospital and sustainability implications

The usage of infusion materials has effects that go beyond clinical safety; they also have effects on the environment and sustainability. Devices made of PVC and DEHP are associated with both insulin adsorption and the release of hazardous plasticizers, which can cause endocrine disruption and long-term damage. Also, a lot of single-use medical plastic waste comes from PVC-based goods. Green hospital initiatives push for a switch to alternatives that do not include DEHP or PVC, which is good for both the environment and the safety of patients. Hospitals that use these kinds of methods have less exposure to dangerous substances for their patients, follow international safety requirements better, and have a smaller impact on the environment. Adopting eco-friendly procurement strategies, using the 5R idea (Reduce, Reuse, Recycle, Recover, Rethink), and putting sustainable medical devices at the top of the list are all important ways to connect clinical safety with larger public health aims (37–42).

Numerous studies have investigated the adherence of insulin to PVC and other infusion materials; yet, significant issues persist. We know many things from lab tests, but real-life clinical trials, especially in newborn and critical care units, are still quite restricted. We also do not have good comparisons between eco-friendly or PVC-free options and regular PVC, so we do not know for sure how safe and effective they are. Another concern that has not been addressed enough is how the loss of preservatives and chemicals impacts the stability of insulin and the safety of patients over time. It is also hard to use the results of studies consistently in practice because the designs and methodologies of the studies are different. Lastly, even while hospitals are working toward greener, more sustainable systems, we still do not know how these changes will lead to improved results for patients or lower dangers from long-term exposure to plasticizers.

## 4 Discussion

**Figure 2:**
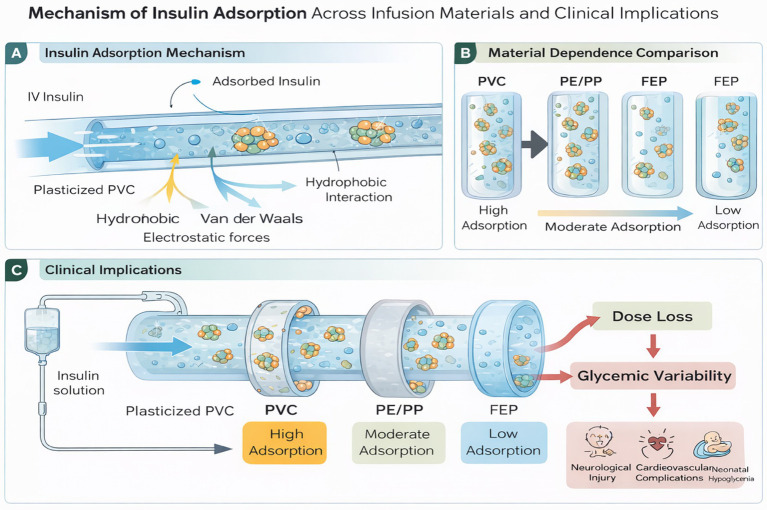
Insulin adsorption across infusion materials. **(A)** Absorption mechanism in PVC. **(B)** Material-dependent adsorption (PVC > PE/PP > FEP). **(C)** Clinical impact: dose loss and glycemic variability.

The goal of this review was to critically examine the issue of insulin adsorption in PVC and alternative infusion materials, with a particular focus on its implications for patient safety, glycemic control, and sustainable hospital practices. By synthesizing evidence from laboratory, modeling, and limited clinical studies, this review highlights both the clinical relevance of adsorption and the broader need to align material choices with safe and environmentally responsible healthcare. The mechanisms and clinical implications of insulin adsorption across infusion materials are summarized in Figure 2.

Schematic illustrating adsorption of insulin onto polymeric infusion materials and its clinical consequences. Adsorption occurs through interfacial interactions between insulin molecules and tubing surfaces and varies according to polymer composition, surface properties, and infusion conditions. Materials such as plasticized PVC exhibit higher adsorption, whereas polyolefins and fluoropolymers demonstrate lower binding affinity. These material-dependent differences may lead to early insulin dose loss, contributing to glycemic variability and potential clinical complications in critical care settings, see Figure 2.

One major theme that emerged is that variations in insulin formulation and delivery systems significantly influence clinical safety and therapeutic efficacy across different patient populations (43). This is consistent with recent intensive care unit (ICU) glycemic control recommendations that advocate continuous intravenous insulin therapy to achieve stable and safe glucose targets in critically ill patients (44). These variations cater to the diverse needs of patients with diabetes, offering tailored solutions that enhance glycemic control, minimize risks, and improve quality of life. Current research highlights the need for more comprehensive studies on insulin infusion sets, glucose-responsive insulin systems, and the physiological implications of insulin delivery methods (45). Non-invasive delivery methods, such as oral insulin and needle-free devices, are being explored to provide more physiological insulin delivery, but these approaches face significant technical and regulatory challenges (46, 47).

The European Association for the Study of Diabetes and the American Diabetes Association have called for more transparency and public availability of adverse event reports related to insulin pumps, which could inform better safety standards and user education (43). The integration of digital health technologies and the development of novel insulin formulations and delivery systems hold promise for addressing these challenges. However, the translation of these innovations into clinical practice requires overcoming regulatory hurdles and ensuring long-term safety and efficacy.

The second theme, Material-Dependent Variability, discusses how the type of material used in infusion systems, the amount of insulin, and the presence of carrier proteins all affect how well insulin is absorbed in infusion mediums. These characteristics can have a big impact on how well and how stable insulin delivery is, which is highly important for controlling blood sugar levels in patients. This risk is especially high in critical care and neonatal settings, when precision glucose control is necessary to prevent life-threatening hypo- or hyperglycemic episodes (8, 25). In contrast, polyethylene (PE), polypropylene (PP), and fluorinated ethylene propylene (FEP) are better options. For example, PE shows almost 100% insulin recovery in the lab (28, 29). But newer options like styrene-ethylene-butylene-styrene (SEBS) and thermoplastic olefins (TPO) nevertheless let meaningful adsorption happen while lowering the risks of plasticizer leaching and environmental damage (29). This variability shows that there are other options besides PVC, but not all of them work equally well to keep insulin stable. More clinical testing is needed before extensive replacement policies can be put into place.

From a physicochemical perspective, insulin adsorption is driven by interfacial interactions between insulin (a surface-active protein) and polymer surfaces, including hydrophobic interactions, van der Waals forces, and depending on surface chemistry—electrostatic contributions. Polymer structure and surface properties (e.g., polarity, surface energy, surface heterogeneity, and roughness) influence both the extent and kinetics of adsorption, which is consistent with recent observations demonstrating that surface chemistry and polymer composition modulate protein adsorption behavior (48). Plasticized PVC may further contribute through microstructural heterogeneity and concomitant sorption of formulation excipients (e.g., phenolic preservatives such as phenol/metacresol), which could indirectly affect insulin stability and delivery consistency. By contrast, polyolefins (PE/PP) and more inert fluoropolymers (e.g., FEP) generally show lower affinity for protein adsorption and/or reduced excipient sorption, offering a plausible mechanistic explanation for the lower insulin losses reported with these materials (49).

Building on this, the third theme, Mitigation Strategies in Practice, demonstrates how healthcare providers can reduce insulin loss regardless of the tubing material used. Priming or preconditioning infusion settings with concentrated insulin solutions before clinical usage is a well-established strategy for saturating binding sites and reducing early adsorption. Studies consistently show that priming with 20 mL or more of insulin solution restores nearly complete recovery, making it one of the most practical and dependable therapies (32, 33). Adjusting flow rates also helps, as larger infusion rates diminish early binding, albeit this is not always possible in neonatal or low-dose conditions (10). Other treatments, such as albumin supplementation, have been attempted, but they create safety issues, especially in newborns, limiting their clinical value (35, 36). Importantly, guidelines now urge routine priming, flushing, and cautious tube material selection as best practices for precise insulin dose and stable glucose control (9).

These two areas have a clear interconnection: whereas material choice determines the baseline risk of insulin adsorption, mitigation measures give clinicians real options to offset these losses. For example, using PE or PP tubing in conjunction with priming techniques can significantly reduce adsorption-related insulin losses and patient safety issues. At the same time, procurement strategies that prioritize low-adsorption, DEHP-free, or non-PVC devices support green hospital activities by lowering harmful plastic use and medical waste (37, 39). However, the paucity of large-scale clinical trials comparing these materials and procedures in real-world ICU and neonatal settings continues to impede consistent deployment. This highlights the importance of interdisciplinary research that combines material science, pharmacology, and critical care practice to develop evidence-based guidelines that protect patients and the environment.

The Green Hospital and Sustainability Implications theme highlights how the use of PVC and DEHP-containing infusion materials poses not only clinical but also environmental risks. The use of PVC and DEHP-containing infusion devices presents a dual challenge: patient safety concerns due to insulin adsorption and environmental hazards arising from toxic plasticizer release and excessive medical waste. Clinically, the adsorption of insulin onto PVC surfaces can compromise glycemic control, increasing the risk of hypo or hyperglycemia in critical care patients. Environmentally, the leaching of DEHP contributes to endocrine disruption, while single-use PVC products exacerbate hospital-generated waste, making healthcare facilities significant contributors to pollution. The implications of these findings extend beyond bedside practice. Hospitals adopting sustainable procurement policies, prioritizing DEHP-free and non-PVC alternatives, directly align with international standards for safer, greener healthcare (37, 39–42).

### 4.1 Implications

The review shows that insulin adsorption on PVC infusion materials is a double-edged sword. It poses clinical hazards such as underdosing, poor glycemic control, and hypoglycemia in ICU and neonatal patients, as well as environmental risks like DEHP leaching and medical plastic waste. This means that the choice of materials has a direct impact on both patient safety standards and hospital procurement strategies. Clinicians should make priming techniques and material selection a part of their everyday job, and governments should push for green hospital reforms to cut down on PVC use and make hospitals more environmentally friendly.

## 5 Conclusion

Insulin adsorption to infusion materials particularly PVC-based systems represents a clinically meaningful yet often underrecognized barrier to accurate glycemic control in critical care and neonatal settings. Evidence synthesized in this review consistently demonstrates that adsorption is influenced by tubing composition, insulin concentration, and infusion conditions, with early infusion periods and low flow rates posing the greatest risk for clinically significant insulin loss. Alternative materials such as polyethylene, polypropylene, and fluoropolymer-based systems generally exhibit lower adsorption profiles, although variability across device architectures indicates that material substitution alone does not universally eliminate the problem. Practical mitigation strategies, especially standardized priming protocols, remain essential regardless of tubing type and should be incorporated into institutional insulin administration guidelines.

Beyond immediate clinical implications, this issue intersects with broader environmental and regulatory considerations. PVC and DEHP-containing medical devices contribute to plasticizer exposure and healthcare-related environmental burdens, supporting the growing rationale for transitioning toward safer, more sustainable alternatives. However, widespread adoption of non-PVC materials faces logistical, economic, and evidentiary challenges, including limited comparative clinical trials, incomplete toxicological data for substitute polymers, and variability in procurement policies across healthcare systems.

Future research should prioritize well-designed clinical studies evaluating real-world insulin delivery accuracy across device types, standardized testing frameworks for adsorption assessment, and integrated evaluations of clinical performance, toxicological safety, and environmental impact. Multidisciplinary collaboration among clinicians, biomedical engineers, pharmacologists, and regulatory agencies will be essential to develop evidence-based standards that optimize both patient safety and sustainability. Addressing insulin adsorption therefore requires not only improved device selection and clinical protocols but also coordinated policy, research, and manufacturing initiatives aimed at safer infusion technologies.

### 5.1 Recommendation

Hospitals and healthcare providers should reduce their use of PVC/DEHP infusion sets by switching to safer options like polyethylene (PE), polypropylene (PP), or fluorinated ethylene propylene (FEP). They should also make sure that infusion lines are always primed with at least 20 mL of insulin solution before use to cut down on early adsorption losses. Clinical teams need to get regular training on the dangers of insulin absorption and the need to follow the right infusion protocols. This is especially important in neonatal and critical care settings. At the policy level, hospital procurement plans should connect with green hospital goals by emphasizing eco-friendly, DEHP-free, and PVC-free devices. This will improve patient safety, optimize glycemic management, and lower the impact on the environment.

## Footnotes

1
www.covidence.org
